# The clinical significance of tumor-infiltrating neutrophils and neutrophil-to-CD8+ lymphocyte ratio in patients with resectable esophageal squamous cell carcinoma

**DOI:** 10.1186/1479-5876-12-7

**Published:** 2014-01-07

**Authors:** Jianbo Wang, Yibin Jia, Nana Wang, Xiaomei Zhang, Bingxu Tan, Guangyu Zhang, Yufeng Cheng

**Affiliations:** 1 Department of Radiation, Qilu Hospital of Shandong University, Jinan 250012, P.R. China

**Keywords:** Intratumoral neutrophils, Neturophil-to-CD8+ lymphocyte ratio, Esophageal squamous cell carcinoma, Immunohistochemistry, Microenvironment

## Abstract

**Background:**

The interaction between tumor cells and inflammatory cells has not been systematically investigated in esophageal squamous cell carcinoma (ESCC). The main aims of the study were to investigate the clinical significance of tumor-infiltrating neutrophils and neturophil-to-CD8+ lymphocyte ratio (NLR), and to analyze the distribution of tumor-infiltrating neutrophils and CD8+ lymphocytes in ESCC treated by curative resection.

**Methods:**

The expressions of CD66b and CD8 were assessed with double staining immunohistochemistry in the surgical specimens from 90 patients with ESCC treated by curative surgery.

**Results:**

We showed that increased intratumoral neutrophils were significantly associated with lymph node metastasis (P = 0.016), and advanced pathological stages (P = 0.013). Decreased peritumoral CD8+ lymphocyte density was more frequently observed in patients with single positive lymph node (p = 0.045). Peritumoral NLR was significantly associated with advanced T stages (p < 0.001), lymph node metastasis (p = 0.041) and a trend towards advanced pathological stages (p = 0.053). Increased intratumoral neutrophils were significantly associated with decreased disease-free survival (p < 0.001) and overall survival (p < 0.001) in univariate analysis and were identified as an independent prognostic factor for disease-free survival (p = 0.006) and overall survival (p = 0.037) in multivariate analysis. Neither the density nor the distribution of tumor-infiltrating neutrophils was significantly correlated with that of CD8+ lymphocytes. The density of intratumoral CD8+ lymphocytes was significantly lower than (P < 0.001) and moderately correlated with (r = 0.434, p < 0.001) that in peritumoral area.

**Conclusions:**

Increased intratumoral neutrophils were an independent poor prognostic factor and peritumoral NLR was significantly associated with disease progression in ESCC treated by curative surgery, suggesting the possible effect of immune misbalance of tumor microenvironment in facilitating ESCC progression. Immunotherapy targeted to the above predictors should be considered in the future.

## Background

Esophageal cancer is the eighth most common cancer type and the sixth leading cause of cancer death worldwide, which was responsible for 482 300 new cases and 406 800 deaths in 2008 [1]. In China, esophageal cancer represents a major health problem, which is the fourth leading cause of cancer death. Squamous cell carcinoma accounts for more than 90% of esophageal cancer cases in Chinese patients [2]. Despite the advancement in diagnosis and treatment modalities, esophageal squamous cell carcinoma (ESCC) still shows a dismal prognosis with a 5-year survival rate less than 15% [3,4]. The current prognostic model is mainly based on pathological parameters such as histological subtype, tumor size and pathological tumor-node-metastasis (pTNM) classification, which are urgently needed to be improved by the integration of new prognostic biomarkers.

Immune reaction was proposed as the seventh hallmark of cancer [5]. The importance of interaction between neoplastic cells and inflammatory cells is becoming increasingly recognized [6]. Leukocyte infiltration is one main characteristic of almost all malignant tumors and the major constituents of these infiltrates include tumor-associated macrophages, neutrophils, mast cells, NK cells, lymphocytes, and so on, but the prognostic value of these infiltrates is still controversial.

Neutrophils, which represent 50%–70% fraction of total circulating leukocytes, make up a significant portion of the leukocyte infiltration in a wide variety of human cancers [7]. Although commonly encountered within tumor microenvironment, neutrophils have not been traditionally considered as a disease modifying entity. Many type of cells within the tumor microenvironment are capable of secreting neutrophil chemotactic substances and neutrophils recruited to tumor site seem to promote cancer cell migration and invasion [8]. Increasing evidence indicated that the presence of neutrophils in tumor tissue be associated with poor prognosis [9-11]. Jensen and colleagues [9] examined the infiltration of neutrophils in 121 localized renal cell carcinomas and showed that the presence of intratumoral neutrophils is an independent poor prognostic factor. Rao et al. [11] provided evidence that increased intratumoral neutrophils be important in the acquisition of a malignant phenotype and predict adverse prognosis in colorectal carcinomas. However, until now, the prognostic effect of tumor-infiltrating neutrophils in ESCC remains unknown.

An elevation in blood neutrophil-to-lymphocyte ratio is considered as a marker of systemic inflammation, which predisposes the tumor to proliferate and metastasize through inhibition of apoptosis, promotion of angiogenesis, and damage of DNA [12-14]. Recent evidence has shown that a high preoperative blood neutrophil-to-lymphocyte ratio was associated with poor outcome in various malignancies undergone potentially curative resection, including esophageal cancer [15,16]. However, the interaction between immune system and tumor cells mostly take place around the tumor tissue. The neutrophil-to-lymphocyte ratio in tumor tissue may also serve as an immune marker of prognostic significance. Cytotoxic CD8+ lymphocytes are crucial components of tumor-specific cellular adaptive immunity and constitute the predominant lymphocyte infiltration in tumor tissue. It is necessary to study the clinical significance of neturophil-to-CD8+ lymphocyte ratio (NLR) in tumor tissue. To date, the prognostic effect of tumor-infiltrating NLR has not been investigated in ESCC.

The main aims of the study were to investigate the clinical significance of tumor-infiltrating (intratumoral and peritumoral) neutrophils and NLR, and to analyze the distribution of tumor-infiltrating neutrophils and CD8+ lymphocytes in ESCC treated by curative resection. The prognostic effect of tumor-infiltrating CD8+ lymphocytes was also studied.

## Materials and methods

### Patients and specimens

Between January 1st and December 31st 2007, 90 patients, who underwent potential radical surgery for ESCC in Department of Thoracic Surgery at Qilu Hospital of Shandong University, were included in the study. Patients were already excluded because of neoadjuvant treatment, perioperative mortality, distant metastasis, stage 0 disease, resection not for curative intent, lost to follow up and lack of tumor tissue. The protocol of the study was approved by the Institutional Ethic Committee of Qilu Hospital of Shandong University. All the patient demographic and clinical data including age, gender, histological grade, lymph node status, pTNM stage, and adjuvant treatment were abstracted from the clinical records. Tumor staging was based on the American Joint Committee on Cancer (AJCC) 6^th^ edition staging manual [17]. A thorough histological examination was made using H&E-stained tissue preparation and the histological grade was determined according to the degree of differentiation of the tumor. Follow-up visits were performed every 3 months for the first 2 years after surgery and thereafter every 6 months up to death or the end of the study (December 2012) for patients without death. At each visit, a clinical history was taken and a physical examination was performed. Routing diagnostic imaging methods included barium meal fluoroscopy and computer tomography. Disease-free survival (DFS) was measured from the date of operation to the date of first evidence of relapse or death, whichever was observed first. For patients who had not relapsed or died, DFS was censored at the last date that the absence of relapse was confirmed. Overall survival (OS) was measured from the date of surgery to the date of death or last follow-up for surviving patients [18].

### Immunohistochemistry

Formalin-fixed paraffin-embedded surgical specimens were used for the immunohistochemical analysis. The presence of available tumor was confirmed by hematoxylin and eosin staining. The tissue blocks were sectioned at 2 μm and mounted on glass slides. Primary antibodies were against CD8 (clone C8/144B, diluted at 1:100, DAKO Corporation Carpenteria, CA, USA) and CD66b (clone G10F5, diluted at 1:200, BD Biosciences, San Jose, CA, USA). Double staining of CD66 and CD8 was carried out with DouSP KIT (Maixin-bio, Jinan, China) to analyze the densities and distributions of CD66+ neutrophils and CD8+ lymphocytes following the manufacturer's protocol with some modification. The sections were deparaffinized in xylene and rehydrated through a graded ethanol series. Antigen retrieval was performed with 0.01 M citrate buffer (pH 6.0) in a pressure cooker for 3 min. Then endogenous peroxidase activity was blocked with endogenous peroxidase blocking solution and non-specific protein-binding sites were blocked through 20 min incubation with non-immune serum at room temperature.

The anti-CD8 antibody was applied for at 37°C for 1.5 h followed by three 5-min washes with PBS. After incubated with biotinylated-labeled second antibody for 30 min, slides were rinsed three times in PBS for 5 min and then incubated in streptavidin alkaline phosphatase complex. After color development with the BCIP/NBT reagent, the sections were washed with PBS, followed by the double-stain amplifier provided with the kit. The anti-CD66b primary antibody was applied at room temperature for 1.5 h, and washed with PBS. Then, the biotinylated-labled second antibody was applied for 30 min followed by streptavidin peroxidase. The immunohistochemical staining was visualized with AEC reagent. Finally, the sections were stained with hematoxylin for 1 min and mounted with an aqueous mounting medium supplied with the kit.

### Evaluation of immunostaining

The analysis was performed with an Olympus IX71S1F-3 Inverted Microscope by two independent observers (Yibin Jia and Nana Wang) blinded to the patients’ clinicopathological details. The average of the values obtained by the two observers was recorded. Intratumoral leukocytes were determined as neutrophils or CD8+ lymphocytes that infiltrated into cancer nests or stroma, whereas peritumoral leukocytes were the cells that distributed along the tumor-myometrial or tumor-connective tissue junction. The tumor sections were screened at low power magnification (×100), and 6 high power fields (×400) with highest number of cell infiltration in either intratumoral or peritumoral areas for neutrophils or CD8+ lymphocytes were selected. The median leukocyte count was used as cutoff to categorize each case into either a high or low group. Areas of necrosis and artifacts and cells within the vessels were omitted. The intratumoral or peritumoral NLR was calculated according to the method of one previous study [19]. NLR was categorized as either ≥ or <1.

### Statistical analysis

Statistical analyses were performed with the SPSS statistical software package (SPSS version 13.0; Chicago, IL). Differences between groups were compared using the chi-square test for categorical variables and the Student t test for continuous variables. Associations between continuous variables were examined by calculating Pearson's correlation coefficient. For univariate analysis, survival curves were obtained with the Kaplan-Meier method and compared by log-rank test. The Cox proportional hazard regression model was used to identify independent prognostic factors. All statistical tests were 2-sided, and P values <0.05 indicated of statistical significance.

## Results

### Patient characteristics

Part of the patient characteristics are listed in Table 1. Median age was 60.5 years (range, 42 to 78 years), and 80% of patients were male. Tumor locations were upper thoracic in 10 patients, middle thoracic in 48 patients, and lower thoracic in 32 patients. The median length of the tumor was 4 cm (range, 0.5-8.5 cm). The histopathological differentiations were poor in 26 cases, moderate in 39 cases, and well in 25 cases. 59 patients (65.6%) had T3/T4 tumors. 33 patients (36.7%) have positive lymph nodes. The pathological stages were stage I in 18 patients, stage II in 41 patients and stage III in 31 patients. 55 patients (61.1%) received surgery alone, 10 (11.1%) received postoperative chemotherapy, 16 (17.8%) received postoperative radiotherapy and 9 (10%) received postoperative chemoradiation. 63 patients (70%) had recurrence and 57 patients (63.3%) died during the follow-up. The estimated 1-, 3-, 5-year DFS and OS rates were 76%, 48%, 36% and 89%, 56%, 41%, respectively. Median DFS was 31.7 months (range, 1.5 to 71.5 months). Median OS was 45.5 months (range, 2.6 to 71.5 months).

**Table 1:** Correlation of intratumoral CD66+ neutrophils, CD8+ lymphocytes and NLR with clinicopathological parameters

	**Total**	**Intratumoral neutrophils**^**a**^			**Intratumoral CD8+ lymphocytes**^**a**^			**Intratumoral NLR**		
	**90**	**Low**	**High**	**P value**	**Low**	**High**	**P value**	**<l**	**≥1**	**P value**
**Gender**										
Male	72	34	38	0.292	36	36	1.0	39	33	0.46
Female	18	11	7		9	9		8	10	
**Age**	42-78									
<60.5^b^	45	25	20	0.292	28	17	**0.02**	21	24	0.291
≥60.5	45	20	25		17	28		26	19	
**Tumor location**										
Upper	10	5	5	1.0	6	4	0.438	6	4	0.858
Middle	48	24	24		21	27		25	23	
Lower	32	16	16		18	14		16	16	
**Tumor length**	0.5-8.5									
<4^b^	52	21	23	0.673	21	23	0.673	20	24	0.209
≥4	38	24	22		24	22		27	19	
**Differential Grade**										
Well	25	14	11	0.764	11	14	0.744	12	13	0.777
Middle	39	19	20		21	18		22	17	
Poor	26	12	14		13	13		13	13	
**T stage**										
1 and 2	31	18	13	0.267	15	16	0.824	17	14	0.719
3 and 4	59	27	32		30	29		30	29	
**LN metastasis**										
Negative	57	34	23	**0.016**	29	28	0.827	30	27	0.919
Positive	33	11	22		16	17		17	16	
**Positive LN**										
0	57	34	23	0.055	29	28	0.968	30	27	0.835
1	12	4	8		6	6		7	5	
≥2	21	7	14		10	11		10	11	
**pTNM stage**										
I	18	12	6	**0.013**	8	10	0.87	12	6	0.343
II	41	24	17		21	20		21	20	
III	31	9	22		16	15		14	17	
**Recurrence**										
**Yes**	63	24	39	**0.001**	32	31	0.818	31	32	0.382
**No**	27	21	6		13	14		16	11	

LN lymph node, pTNM pathological tumor node metastasis, NLR, CD66+ neutrophil-to-CD8+ lymphocyte ratio, values in bold signify p < 0.05, ^a^Grouped by median, ^b^The median.

### Analysis of immunohistochemical parameters

**Figure 1:**
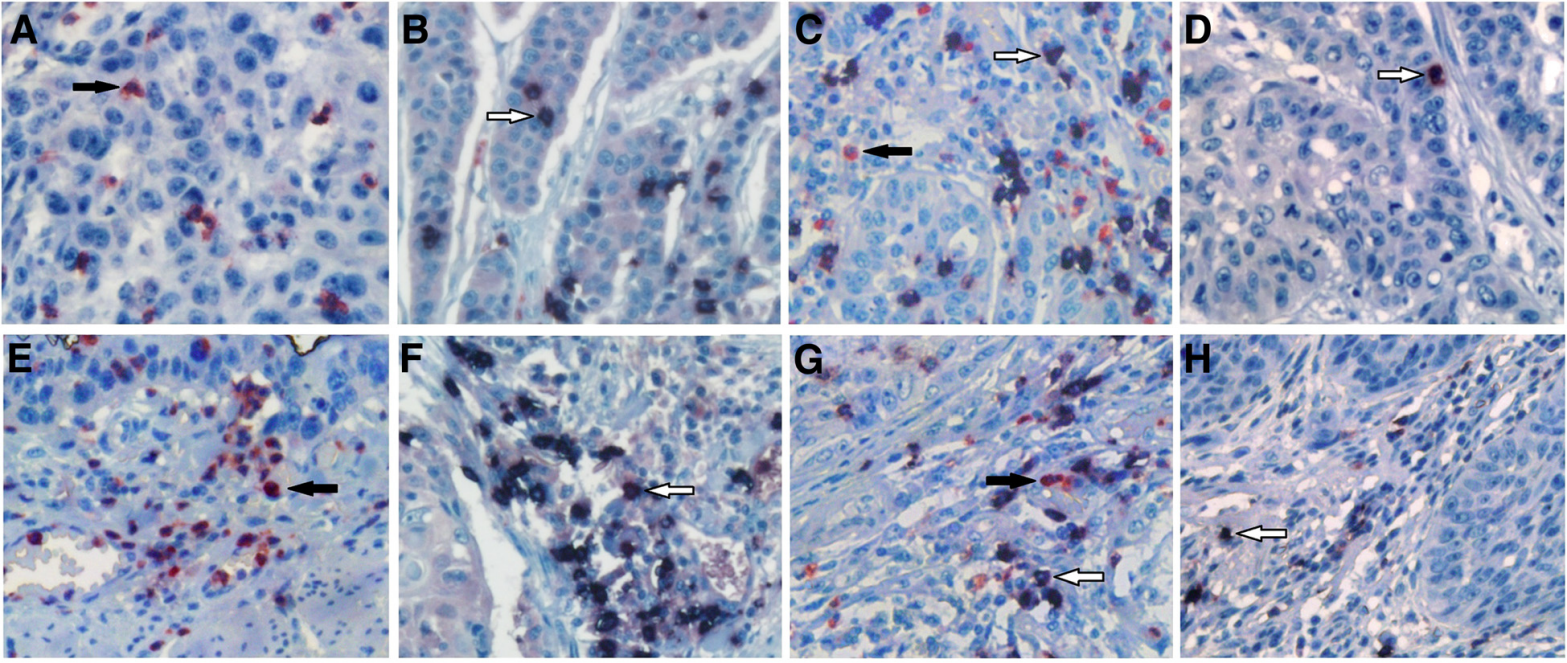
**Representative examples of immunostaining of CD66b + neutrophils (in red) or CD8+ lymphocytes (in black) from intratumoral (A, B, C, D) or peritumoral (E, F, G, H) areas of esophageal squamous cell carcinoma (x400). A**, **E** infiltrated mainly by CD66b + neutrophils (black arrow); **B**, **F** infiltrated mainly by CD8+ lymphocytes (white arrow); **C**, **G** infiltrated by both CD66b + neutrophils and CD8+ lymphocytes; **D**, **H** infiltrated by low density of the inflammatory cells.

Representative images of neutrophil and CD8+ lymphocyte infiltration are shown in Figure 1. The median densities of intratumoral and peritumoral neutrophils were 18.5 cells/HPF (range, 0–387 cells/HPF) and 19 cells/HPF (range, 0–247 cells/HPF), respectively. The median densities of intratumoral and peritumoral CD8+ lymphocytes were 19 cells/HPF (range, 0–122 cells/HPF) and 32 cells/HPF (range, 0–200 cells/HPF), respectively. Infiltration of neutrophils or CD8+ lymphocyte was identified in both stroma and islet of the tumor, and there were no marked differences in neutrophil or CD8+ lymphocyte density between different intratumoral areas. The density of intratumoral neutrophils was not significantly different from that of peritumoral areas (p = 0.348). The density of intratumoral CD8+ lymphocytes was significantly lower than that of peritumoral area (p < 0.001). The distribution of neutrophils was not associated with that of CD8+ lymphocytes. The density of intratumoral neutrophils were not significantly associated with that of intratumoral CD8+ lymphocytes (r = 0.005, p = 0.966), and the density of neutrophils in peritumoral areas were not significantly correlated with that of CD8+ lymphocytes in peritumor (r = −0.127, p = 0.232), either. The density of intratumoral neutrophil was not significantly correlated with that in peritumor (r = 0.202, p = 0.056). The density of intratumoral CD8+ lymphocytes was moderately correlated with that in peritumoral area (r = 0.434, p < 0.001). For patients with lymph node metastasis, CD8+ lymphocyte density in intratumoral area was also significantly correlated with that in peritumor (r = 0.55, p = 0.001).

### Correlation of immunohistochemical parameters with clinicopathological parameters

As is shown in Tables 1 and 2, increased intratumoral neutrophils was significantly associated with lymph node metastasis (P = 0.016), advanced pTNM stages (P = 0.013) and recurrence (p = 0.001). High intratumoral CD8+ lymphocytes were more frequently observed in elder patients (p = 0.02). Intratumoral NLR was not associated with any of the clinicopathological parameters. Peritumoral neutrophil density was not associated with the studied clinicopathological parameters. Decreased peritumoral CD8+ lymphocyte density was more frequently observed in patients with single positive lymph node (p = 0.045). Increased peritumoral NLR was associated with male gender (p = 0.009), advanced T stages (p < 0.001), positive lymph node metastasis (P = 0.041) and a trend towards advanced pathological stage (p = 0.053).

**Table 2:** Correlation of peritumoral CD66+ neutrophils, CD8+ lymphocytes and NLR with clinicopathological parameters

	**Total**	**Peritumoral neutrophils**^**a**^			**Peritumoral CD8+ lymphocytes**^**a**^			**Peritumoral NLR**		
	**90**	**Low**	**High**	**P value**	**Low**	**High**	**P value**	**<l**	**≥1**	**P value**
**Gender**										
Male	72	38	34	0.292	37	35	0.598	40	32	**0.009**
Female	18	7	11		8	10		16	2	
**Age**	42-78									
<60.5^b^	45	25	20	0.292	25	20	0.292	27	18	0.664
≥60.5	45	20	25		20	25		29	16	
**Tumor location**										
Upper	10	6	4	0.612	6	4	0.769	6	4	0.634
Middle	48	25	23		24	24		28	20	
Lower	32	14	18		15	17		22	10	
**Tumor length**	0.5-8.5									
<4.0^b^	52	18	26	0.092	20	24	0.399	30	14	0.254
≥4.0	38	27	19		25	21		26	20	
**Differential grade**										
Well	25	9	16	0.185	14	11	0.764	12	13	0.221
Middle	39	20	19		19	20		26	13	
Poor	26	16	10		12	14		18	8	
**T stage**										
1 and 2	31	18	13	0.267	12	19	0.12	27	4	**<0.001**
3 and 4	59	27	32		33	26		29	30	
**LN metastasis**										
Negative	57	27	30	0.512	26	31	0.274	40	17	**0.041**
Positive	33	18	15		19	14		16	17	
**Positive LN**										
0	57	27	30	0.764	26	31	**0.045**	40	17	**0.014**
1	12	7	5		10	2		3	9	
≥2	21	11	10		9	12		13	8	
**pTNM stage**										
I	18	9	9	0.972	8	10	0.536	13	5	0.053
II	41	21	20		19	22		29	12	
III	31	15	16		18	13		14	17	
**Recurrence**										
**Yes**	63	31	32	0.818	32	31	0.818	38	25	0.569
**No**	27	14	13		13	14		18	9	

LN lymph node, pTNM pathological tumor node metastasis, NLR CD66+ neutrophil-to-CD8+ lymphocyte ratio, values in bold signify p < 0.05, ^a^Grouped by median, ^b^The median.

### Survival analysis

**Figure 2:**
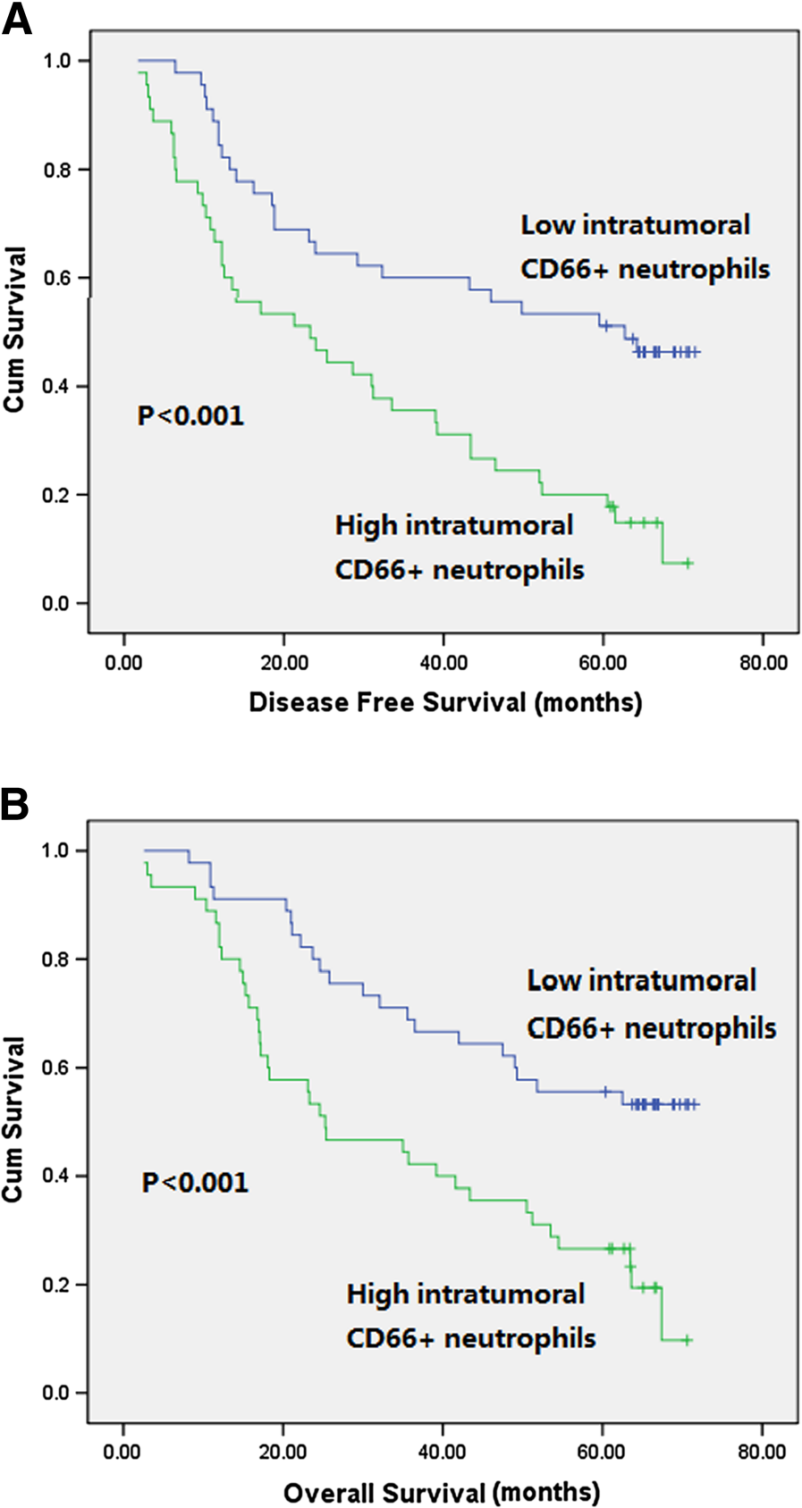
**Kaplan-Meier analysis of intratumoral CD66b + neutrophils of 90 patients with esophageal squamous cell carcinoma.** Increased intratumoral neutrophils were significantly associated with decreased disease-free survival **(A)** and overall survival **(B)**.

To identify variables of potential prognostic significance in the patients with ESCC, univariate and multivariate analyses were employed to investigate the impact of tumor-infiltrating neutrophils, CD8+ lymphocytes, NLR and other clinicopathological parameters on the prognosis of the 90 ESCC patients. In univariate analysis, increased intratumoral neutrophils were significantly associated with poor DFS (p < 0.001) and OS (p < 0.001) (Figure 2). No prognostic significance was found for intratumoral and peritumoral CD8+ lymphocytes, intratumoral and peritumoral NLR and peritumoral neutrophils. The 5-year DFS and OS rates for patients with increased intratumoral CD66+ neutrophils were 20% and 26.7%, compared with 51.1% and 55.5% for patients with decreased intratumoral CD66+ neutrophils, respectively. Clinicopathological factors significantly associated with short DFS were moderate differentiation, pT4 stage, lymph node metastasis and advanced pTNM stages. Variables significantly associated with poor OS were advanced T stages, lymph node metastasis, advanced pTNM stages and adjuvant radiation (Table 3). Multivariate analysis revealed that upper tumor location, advanced pTNM stages and increased intratumoral neutrophils were independently associated with decreased DFS and advanced pTNM stages, postoperative radiation, and increased intratumoral neutrophils were independent predictors for poor OS (Table 4).

**Table 3:** Univariate analysis of clinicopathological and immunohistochemical parameters

**Univariate analysis**	**Disease-free survival**		**Overall survival**	
	**HR (95% CI)**	**P value**	**HR (95% CI)**	**P value**
**Gender**	1.182 (0.64-2.185)	0.593	1.154 (0.608-2.19)	0.661
**Age**	1.092 (0.665-1.793)	0.727	1.27 (0.753-2.144)	0.37
**Tumor location**				
Upper	Ref.	0.213	Ref.	0.6
Middle	0.517 (0.2554-1.202)	0.083	0.653 (0.286-1.493)	0.312
Lower	0.553 (0.545-1.608)	0.135	0.698 (0.296-1.65)	0.413
**Tumor length**	1.172 (0.715-1.923)	0.529	1.343 (0.797-2.262)	0.269
**Differential Grade**				
Well	Ref.	0.095	Ref.	0.17
Moderate	1.947 (1.045-3.629)	**0.036**	1.881 (0.971-3.645)	0.061
Poor	1.334 (0.666-2.672)	0.417	1.449 (0.697-3.015)	0.321
**T stage**				
T1	Ref.	0.121	Ref.	**0.026**
T2	1.359 (0.539-3.454)	0.519	1.425 (0.477-4.253)	0.526
T3	1.954 (0.871-4.385)	0.104	2.706 (1.061-6.9)	**0.037**
T4	3.328 (1.113-9.951)	**0.031**	4.618 (1.404-15.196)	**0.012**
**LN metastasis**	2.506 (1.51-4.157)	**<0.001**	2.469 (1.455-4.188)	**0.001**
**Positive LN number**				
0	Ref.	**0.001**	Ref.	**0.002**
1	3.479 (1.756-6.89)	**<0.001**	3.01891.491-6.112	**0.002**
≥2	2.117 (1.176-3.812)	**0.012**	2.20391.195-4.063	**0.011**
**pTNM stage**				
I	Ref.	**<0.001**	Ref.	**<0.001**
II	2.03 (0.919-4.482)	0.08	2.334 (0.951-5.729)	0.064
III	4.365 (1.962-9.713)	**<0.001**	5.254 (2.139-12.91)	**<0.001**
**Treatment regimens**				
Surgery alone	Ref.	0.081	Ref.	0.063
Adjuvant chemotherapy	0.839 (0.354-1.993)	0.692	0.747 (0.291-1.919)	0.545
Adjuvant radiation	1.821 (0.961-3.451)	0.066	1.99 (1.04-3.807)	**0.038**
Adjuvant chemoradiation	2.152 (0.992-4.67)	0.053	2.054 (0.902-4.676)	0.086
**Immunohistochemical markers**				
Intratumoral neutrophils	2.505 (1.496-4.196)	**<0.001**	2.559 (1.487-4.406)	**<0.001**
Intratumoral CD8+ lymphocytes	0.896 (0.546-1.471)	0.665	0.959 (0.57-1.613)	0.875
Intratumoral NLR	1.279 (0.78-2.097)	0.329	1.316 (0.783-2.214)	0.3
Peritumoral neutrophils	1.035 (0.63-1.7)	0.893	1.119 (0.664-1.886)	0.672
Peritumoral CD8+ lymphocytes	0.952 (0.581-1.561)	0.846	1.032 (0.613-1.735)	0.907
Peritumoral NLR	1.306 (0.788-2.165)	0.3	1.292 (0.761-2.195)	0.343

LN lymph node, pTNM pathological tumor node metastasis, NLR CD66+ neutrophil-to-CD8+ lymphocyte ratio, HR hazard ratio, CI confidence interval, Ref. reference, Values in bold signify p < 0.05.

**Table 4:** Independent predictors for survival by multivariate analysis

**Multivariate analysis**	**Category compared**	**HR (95% CI)**	**P value**
Disease-free survival			
Tumor location	Upper vs. middle and lower	0.474 (0.228-0.987)	0.046
pTNM stage	I, II vs. III	1.892 (1.104-3.241)	0.02
Intratumoral neutrophils	<18.5 vs. ≥18.5	2.174 (1.249-3.784)	0.006
Overall survival			
pTNM stage	I, II vs. III	2.122 (1.205-3.736)	0.009
Treatment regimens	Surgery alone vs. Adjuvant radiation	1.855 (1.061-3.244)	0.03
Intratumoral neutrophils	<18.5 vs. ≥18.5	1.858 (1.038-3.325)	0.037

pTNM pathological tumor node metastasis, HR hazard ratio, CI confidence interval.

## Discussion

Inflammation seems to play a crucial role in cancer development by promoting or restraining progression, angiogenesis and metastasis, and impacting response to systemic therapies [20]. Neutrophils and lymphocytes, which constitute the predominant proportion of total circulating leukocytes, play a key role in host systemic immune response, but their effect on tumor progression should mostly be realized in the tumor microenvironment. In the present study, we utilized the methods of double staining immunohistochemistry to investigate the prognostic value of tumor-infiltrating neutrophils, CD8+ lymphocytes and NLR and to analyze the distribution of tumor-infiltrating neutrophils and CD8+ lymphocytes in ESCC treated by curative resection. Our study firstly established that increased intratumoral neutrophils were significantly correlated with lymph node metastasis and advanced pTNM stages, and increased peritumoral NLR was significantly associated with depth of invasion, lymph node metastasis and a trend towards advanced pathological stages in ESCC treated by curative resection. Moreover, univariate and multivariate analyses revealed that increased intratumoral neutrophils were a prognostic factor for poor DFS and OS, independent of certain well-established clinical features, including depth of invasion, lymph node metastasis, and pTNM stages.

Neutrophils are short-lived white blood cells derived from bone marrow myeloid precursors. Attention has long been focused on their short-term antimicrobial and tissue-damaging function. However, growing evidence recently suggests that neutrophils play an important role in host’s reaction to cancer [21]. The presence of tumor-associated neutrophils have been demonstrated to be associated with poor clinical outcomes of several malignancies including clear cell renal cell carcinoma [9], gastric cancer [22], colorectal cancer [11], and hepatocellular carcinoma [10]. The present study firstly investigated the clinical significance of tumor infiltrating neutrophils in ESCC. Our results revealed that intratumoral neutrophils be a significant prognostic factor in ESCC treated by curative surgery, which was consistent with the results of previous studies in other tumors.

Currently, the exact role of neutrophils in cancer initiation, progression and metastasis remains unclear. It has been hypothesized that tumor-associated neutrophils may have different states of differentiation which can thus take an anti-tumorigenic (“N1-phenotype”) versus a pro-tumorigenic (“N2”) phenotype [23]. The states of neutrophils in tumor tissue were recognized by expression of immuno-activating cytokines and chemokines, and capability of killing tumor cells. In models of lung cancer, infiltrating neutrophils are driven by TGF-b to acquire a protumor phenotype. The phagolysosomes of neutrophils contain several enzymes capable of reducing molecular oxygen into superoxide radicals. Reactive oxygen species from neutrophils were reported to exert both genotoxic effects and carcinogenesis [24]. Neutrophils could also release a group of proteinases including neutrophil elastase, matrix metalloproteinase-8, and matrix metalloproteinase-9 to process and degrade a wide range of cytokines, chemokines, and their cognate receptors, in addition to their well-described ability to remodel extracellular matrix and enhance angiogenesis, which could promote the invasion and metastasis of cancer cells [25-27].

It is well recognized that cytotoxic CD8+ lymphocytes constitute one of the most important effector mechanisms of anti-tumor immunity [28]. In both animal models and humans, CD8+ lymphocytes have been shown to play an important role in the host's defense against malignancies, and most cancer vaccine strategies have focused on the induction of effector CD8+ lymphocytes that kill tumor cells [29]. It has been shown that a high infiltration of CD8+ lymphocytes be associated with good clinical outcome in several types of human cancer, including cancer of esophagus [30-33]. However, other studies did not show the clinical significance of tumor infiltrated CD8+ lymphocytes [34]. The inconsistent results in different studies could be explained by discrepancies in sample size, study population, treatment modality, and pathological type. In our study, we did not found any association between intratumoral or peritumoral CD8+ lymphocyte infiltration and clinicopathological parameter such as depth of invasion, pathological stages, or clinical outcome. Only when analyzed by positive lymph node number, low peritumoral CD8+ lymphocytes were associated with one lymph node metastasis. Tumor-infiltrating CD8+ lymphocytes could not predict progression or prognosis in ESCC treated by curative resection. Our results indicated that the antitumor effect of CD8+ lymphocytes could be partly impaired in the microenvironment of esophageal squamous cell carcinoma, especially in the intratumoral area. The impairment may be mediated by the following mechanisms: immunosuppressing factors released by tumor cells, deficient tumor antigen presentation by dendritic cells, reduced production of costimulating cytokines by helper CD4+ T-cells, and recruitment of regulatory immune cells by tumor cells [35].

Recently, great interests have been generated in elucidating the role of cancer inflammation in predicting disease load and prognosis. Blood neutrophil to lymphocyte ratio has been found to be associated with disease progression in a number of malignancies [36]. However, the systemic immune cells must infiltrate the tumor tissue to function their antitumor or protumor effect. Cancer cells recruit inflammatory cells, including neutrophils, which can suppress the action of cytotoxic lymphocytes. Cancer cells themselves may secrete various molecules to induce a tumor facilitating cytotoxic lymphocytes. Currently, evidence is limited about the prognostic effect of tumor-infiltrating NLR in malignancies. Marius et al. [19] assessed the prognostic impact of intratumoral NLR in resectable non-small cell lung cancer and found it as an independent prognostic factor for a high rate of disease recurrence and poor survival. Another study showed that elevated intratumoral NLR was significantly associated with decreased DFS and OS in hepatocellular carcinoma following resection [10]. In the present study, we firstly analyzed the clinical significance of both intratumoral and peritumoral NLRs in ESCC treated by curative surgery. Although no prognostic effect of intratumoral or peritumoral NLR was shown, we interestingly found that increased peritumoral NLR was associated with depth of invasion, lymph node metastasis and a trend towards advanced pathological stages. In the peritumoral region of ESCC, infiltrating neutrophils may suppress the cytotoxic response of CD8+ lymphocytes and thereby allow tumor cells to evade immune surveillance. The results are in agreement with recent findings showing that, in mice, naturally activated neutrophils in the tumor microenvironment were mainly of the polarized N2 phenotype promoting tumor progression, partly associated with suppression of CD8+ lymphocytes [23]. However, this does not preclude the possibility that peritumoral microenvironment with high neutrophil and low CD8+ lymphocyte infiltration is more suitable for tumor invasion and both immune cells do not act on each other directly.

Human tumor tissue can be anatomically classified into areas of intratumor and peritumor, each with distinct compositions and functional properties. The density of intratumoral neutrophil was not significantly different from or correlated with that of peritumoral area, whereas the density of intratumoral CD8+ lymphocytes was significantly lower than and moderately correlated with that of peritumoral area. Infiltrating of intratumoral CD8+ lymphocyte may partly determined by recruitment of the lymphocytes in the peritumoral region. Intratumoral environment usually exhibited a generalized immunosuppressive status, resulting in a relatively low density of intratumoral cytoxic CD8+ lymphocytes. We observed great discrepancies in the amount of both neutrophil and CD8+ lymphocyte infiltration between different patients. The mechanism why patients may be associated with mild or extensive level of inflammatory cells remains unclear. In addition, it has been found that the density of intratumoral neutrophils was significantly correlated with that of CD8+ lymphocytes in resected hepatocellular cell carcinoma [10]. Neutrophil depletion could result in more activated CD8+ lymphocytes intratumorally [23]. However, in our study, neither the distribution nor the density of neutrophils was correlated with that of CD8+ lymphocytes in ESCC, which suggest that the recruitment of the two types of inflammatory cells may represent different characteristic of ESCC that do not correlated directly.

## Conclusions

In conclusion, we showed that increased intratumoral CD66+ neutrophils were an independent prognostic factor for poor DFS and OS in ESCC treated by curative surgery. Moreover, we demonstrated that increased peritumoral NLR was a predictor for advanced T stages, lymph node metastasis and a trend towards advanced disease stages. This indicated that the progression of ESCC may be governed at least in part by the state of the local innate immune response. This might also aid the clinician to select a suitable therapy for the individual patients, e.g. favoring a more aggressive regimen in tumors with an increased intratumoral neutrophils or misbalance of peritumoral neutrophil and CD8+ lymphocytes. Moreover, Immunotherapy targeted to the above predictors may also help improve the prognosis of esophageal cancer.

## Abbreviations

ESCC: Esophageal squamous cell carcinoma; NLR: Neutrophils and neturophil-to-CD8+ lymphocyte ratio; DFS: Disease-free survival; OS: Overall survival.

## Competing interests

The authors declare that they have no competing interest.

## Authors’ contributions

WJB carried out experiments, analyzed the data, and participated in the study design and manuscript writing. JYB and WNN contributed to data analyzing; ZXM, TBX and ZGY collected data, and participated in experiment performance. CYF designed experiments and wrote the manuscript. All authors read and approved the final version of the manuscript.
